# Comparison of Qualitative and Quantitative Methods for Gingival Thickness Assessment: Color-Coded Probe Versus Ultrasound Measurements

**DOI:** 10.3390/ma19050893

**Published:** 2026-02-27

**Authors:** Jakub Hadzik, Paweł Kubasiewicz-Ross, Tomasz Gedrange, Krzysztof Kujawa, Marzena Dominiak

**Affiliations:** 1 Department of Dental Surgery, Faculty of Dentistry, Wroclaw Medical University, Krakowska 26, 50-425 Wroclaw, Poland; 2 Department of Orthodontics, Carl Gustav Carus University Hospital Dresden, Technische Universität Dresden, D-01307 Dresden, Germany; 3 Statistical Analysis Centre, Wroclaw Medical University, K. Marcinkowskiego 2-6, 50-368 Wroclaw, Poland

**Keywords:** gingival thickness, gingival phenotype, color-coded periodontal probe, ultrasonography, non-invasive measurement, soft tissue assessment, peri-implant tissues

## Abstract

Accurate assessment of gingival thickness is essential for clinical decision-making in implant dentistry, periodontology, and orthodontics. Qualitative phenotype assessment using color-coded periodontal probes is commonly applied as a rapid screening tool, whereas ultrasonography provides quantitative, non-invasive measurements expressed in millimeters. The relationship between these two approaches, however, remains insufficiently defined. The aim of this study was to evaluate the correspondence between qualitative gingival phenotype classification using a color-coded probe and quantitative soft tissue thickness measured with an ultrasound-based system. In this observational diagnostic substudy, the gingival phenotype was categorized as thin, medium, thick, or very thick based on probe translucency and compared with ultrasound-derived thickness values obtained at standardized sites. Qualitative phenotype categories showed a consistent association with quantitative measurements, supporting the role of color-coded probes as screening tools within a complementary diagnostic framework. Ultrasonography provided objective data suitable for detailed assessment and monitoring. These findings support a complementary diagnostic approach combining qualitative screening with quantitative measurement.

## 1. Introduction

Gingival phenotype represents a clinically relevant biological parameter affecting soft tissue stability, esthetic outcomes, and susceptibility to recession [1,2]. Thin phenotypes are associated with increased risk of soft tissue collapse and recession following restorative or surgical procedures, whereas thicker phenotypes demonstrate greater dimensional stability [3,4,5]. Therefore, reliable phenotype assessment is essential for risk stratification and individualized treatment planning.

The availability of a simple, non-invasive, and clinically feasible screening method for soft tissue thickness assessment is of growing importance across multiple dental disciplines. Such an approach would enable not only routine chairside evaluation but also repeated measurements over time, facilitating patient monitoring and supporting qualification for various treatment modalities. In particular, non-invasive screening tools may play a key role during the initial diagnostic phase, where soft tissue phenotype assessment contributes to risk stratification and treatment planning.

A variety of methods for oral and peri-implant soft tissue thickness assessment have been described in the literature. These include transgingival probing without incision, direct measurements performed after surgical exposure, radiographic assessment using cone-beam computed tomography (CBCT), qualitative evaluation with color-coded periodontal probes, and non-invasive ultrasound-based techniques [6,7,8,9,10,11,12,13,14].

When selecting non-invasive methods for soft tissue thickness assessment, both qualitative and quantitative approaches are available. Qualitative gingival phenotype assessment based on probe translucency has been widely adopted in clinical practice due to its simplicity and non-invasive character. Color-coded periodontal probes have been introduced to further standardize this approach by enabling the classification of soft tissues into multiple phenotype categories. In contrast, ultrasonography provides quantitative measurements of soft tissue thickness expressed in millimeters.

Given the coexistence of these qualitative and quantitative approaches, an important clinical question arises as to whether they can be used interchangeably or should instead be regarded as complementary tools serving different diagnostic purposes. Importantly, the purpose of comparing these approaches is not to replace qualitative chairside assessment with digital techniques, but to provide scientific validation for a widely used screening method and to define a complementary diagnostic framework in which rapid phenotype classification is integrated with objective quantitative measurement when higher precision is required. While qualitative techniques, such as color-coded periodontal probes, are designed for rapid chairside phenotype classification, quantitative methods provide numerical values that may be required for detailed treatment planning, longitudinal monitoring, or research applications [5,15,16].

From a clinical perspective, the selection of an appropriate assessment method should depend on the intended use, including screening, patient qualification for specific procedures, longitudinal follow-up, or standardized data collection. However, the extent to which qualitative phenotype classification corresponds to clinically meaningful quantitative thickness ranges remains insufficiently established. Without such validation, the diagnostic accuracy of qualitative assessment methods and their ability to reliably reflect underlying tissue dimensions cannot be fully determined.

Therefore, direct comparison of qualitative and quantitative measurement approaches, together with evaluation of their level of relationship, is essential for informed clinical decision-making. By assessing the correspondence between color-coded probe-based phenotype classification and ultrasound-derived soft tissue thickness values, it becomes possible to estimate the accuracy of the qualitative method, identify its limitations, and define clinical scenarios in which each approach may be most appropriate. Such comparative evaluation may assist clinicians in selecting the most suitable assessment strategy based on the required level of precision, invasiveness, and feasibility in everyday practice.

The aim of the present study was to evaluate whether qualitative gingival biotype assessment performed using a color-coded periodontal probe provides clinically meaningful approximation of ultrasound-derived soft tissue thickness, using ultrasound-based measurements as a reference method.

## 2. Materials and Methods

### 2.1. Study Design

The present study was designed as a non-interventional observational diagnostic substudy conducted within the organizational and ethical framework of an investigator-initiated randomized controlled clinical trial evaluating peri-implant soft tissue thickness in the esthetic zone.

Although the data were collected from patients associated with a randomized clinical trial, the present analysis was designed to evaluate the correspondence between qualitative probe-based phenotype assessment and quantitative ultrasound measurements of peri-implant soft tissue thickness and did not evaluate any treatment-related outcomes. Treatment allocation and clinical outcomes of the parent randomized clinical trial were not considered in the present analysis.

The study population consisted of consecutive patients presenting for screening and eligibility assessment for participation in the parent clinical trial prior to randomization; not all screened patients were ultimately enrolled in the randomized study.

All measurements included in this substudy were non-invasive, performed during the qualification visit, and conducted with the patients’ informed consent. No additional study visits or procedures beyond those defined in the parent study protocol were required.

### 2.2. Ethical Approval

The study protocol, patient information sheet, and informed consent templates for the parent randomized clinical trial were reviewed and approved by the Bioethics Committee of Wrocław Medical University (Poland) (approval number: KB-863/2021; date of approval: 28 October 2021). Following ethical approval, permission to conduct the study was obtained, and the trial was carried out at the study site. The parent clinical trial was registered in the ClinicalTrials.gov database (Study Identifier: NCT07324187).

The study constitutes a predefined, non-interventional observational diagnostic substudy conducted during the pre-randomization screening and qualification phase of the registered trial. All procedures included in this substudy were non-invasive and did not involve assignment to therapeutic interventions.

All research procedures were performed in accordance with the ethical principles of the Declaration of Helsinki, applicable European regulations, and relevant local regulatory requirements.

### 2.3. Study Population

The study population consisted of patients presenting for screening and eligibility assessment for participation in the parent randomized clinical trial. Importantly, inclusion in the present substudy was independent of final inclusion in the randomized clinical trial. Soft tissue thickness measurements were performed in patients attending the qualification visit, regardless of whether they were ultimately randomized or received any interventional treatment. No additional inclusion or exclusion criteria were applied for the purposes of this substudy.

### 2.4. Measurement Site

Soft tissue thickness was assessed at a tooth adjacent to the edentulous site. In cases of a missing maxillary lateral incisor (e.g., tooth 22), measurements were performed on the mesial aspect of the neighboring tooth (tooth 21). All measurements were performed by a single trained and calibrated examiner, following standardized protocols and in accordance with the manufacturers’ recommendations for the use of the measurement devices [17]. Color-coded probe was inserted into the gingival sulcus to a depth of approximately 2 mm during translucency assessment; ultrasound measurements were obtained at the corresponding sulcular level.

### 2.5. Clinical Biotype Assessment Using a Color-Coded Probe

**Figure 1:**
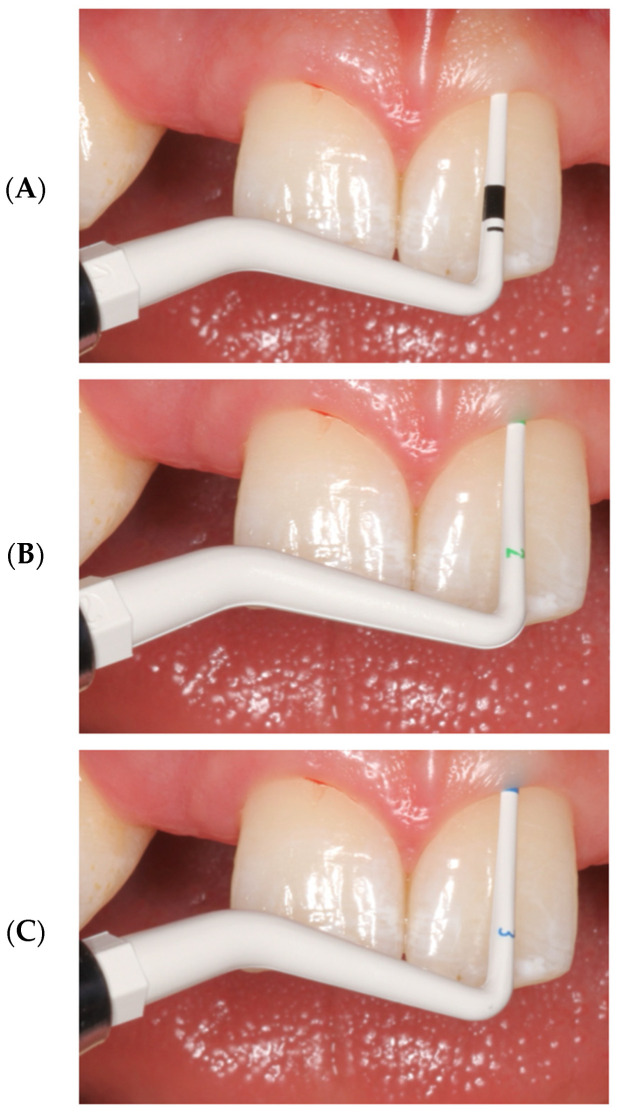
Stepwise clinical assessment of gingival phenotype using a color-coded periodontal probe at a tooth adjacent to the edentulous site. The probe was inserted into the sulcus and evaluated for translucency through the gingival tissue using sequential colors: (**A**) white, (**B**) green, and (**C**) blue. The most intense color visible through the tissue determined the biotype category (thin, medium, thick, or very thick).

Initial assessment of gingival phenotype was performed using a color-coded periodontal probe based on visual translucency of the probe through the gingival tissue. Colorvue Biotype Probe System (Hu-Friedy, Chicago, IL, USA) was used in this study. This commercially available color-coded periodontal probe features sequentially colored segments of increasing diameter, enabling stratification of gingival tissues based on visual probe translucency through the soft tissue. Gingival phenotype was qualitatively stratified into four categories (thin, medium, thick, and very thick) according to the color visible through the soft tissue at the measurement site (Figure 1). This assessment was performed by an experienced clinician and recorded immediately after probing.

The four-category qualitative stratification applied in the present study follows the methodological approach proposed by Fischer et al. [15] for color-coded probe assessment, while its clinical interpretation is aligned with the simplified thin–thick framework recommended by the 2017 World Workshop on the Classification of Periodontal and Peri-Implant Diseases and Conditions.

Visibility of the white probe tip → thin phenotype.

Visibility of the green probe tip → medium phenotype.

Visibility of the blue probe tip → thick phenotype.

No visibility of any probe color → very thick phenotype.

### 2.6. Ultrasonic Measurement of Soft Tissue Thickness

**Figure 2:**
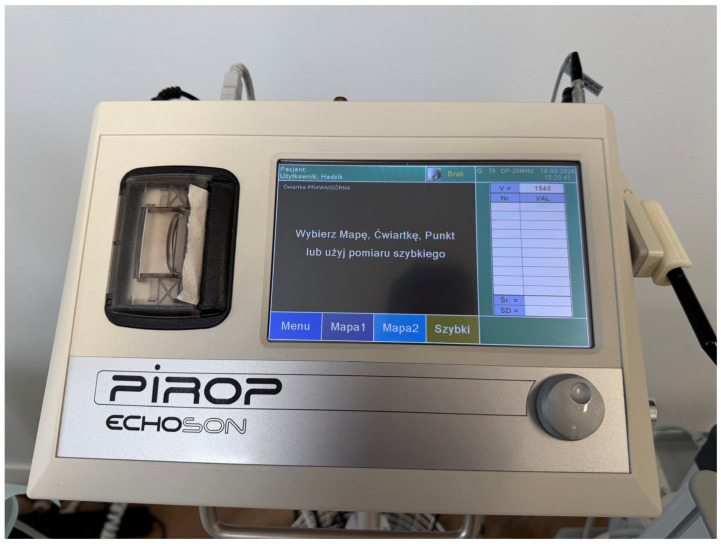
Pirop ultrasound-based biometric scanner (ECHOSON^®^, Poland) used for non-invasive quantitative assessment of peri-implant soft tissue thickness. Ultrasonic measurements were performed according to a standardized protocol and provided soft tissue thickness values expressed in millimeters.

Subsequently, at the same measurement site, the same single trained and calibrated examiner quantitatively assessed soft tissue thickness using a non-invasive ultrasonic device (Pirop^®^, ECHOSON^®^, Pulawy, Poland). Ultrasonic measurements yielded quantitative soft tissue thickness values expressed in millimeters (Figure 2).

For each site, the Pirop^®^ system automatically acquired ten consecutive ultrasonic readings without repositioning of the probe. The mean value of these ten measurements, calculated by the device software, was used for further analysis in order to minimize random measurement error and enhance reproducibility.

Each patient contributed one standardized measurement site located at the tooth adjacent to the edentulous space, on the mesial aspect of the neighboring tooth, resulting in a total of 80 measurement sites.

**Figure 3:**
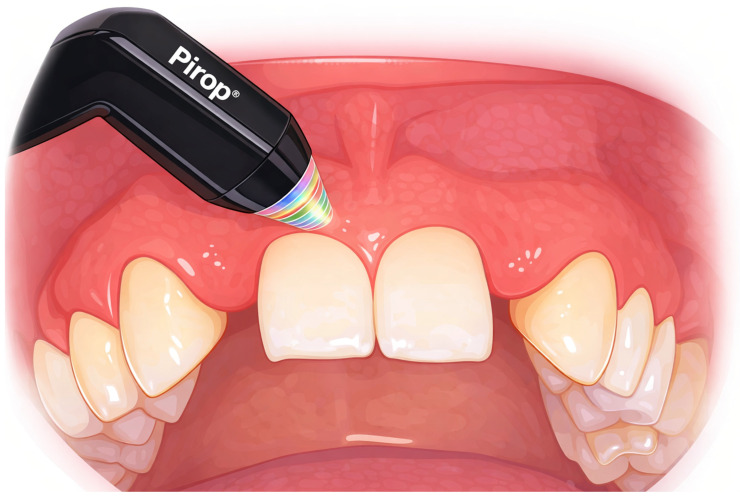
Conceptual illustration of gingival quantitative, non-invasive ultrasonic measurement of gingival thickness performed with the Pirop.

At each site, gingival thickness was first assessed qualitatively using the Colorvue biotype probe, followed by quantitative measurement using the Pirop ultrasound-based system (Figure 3). Thus, 80 paired measurements were obtained, consisting of qualitative gingival phenotype classification and corresponding quantitative soft tissue thickness values.

The Pirop ultrasonic system has been previously evaluated in Hadzik et al.’s [18,19] clinical studies, demonstrating acceptable repeatability and reliability for non-invasive assessment of gingival soft tissue thickness.

### 2.7. Statistical Analysis

Statistical analysis was performed to evaluate the relationship between qualitative gingival phenotype classification obtained using the Colorvue biotype probe and quantitative gingival thickness values measured with the Pirop ultrasound-based system.

Descriptive statistics were calculated for ultrasonic gingival thickness measurements within each Colorvue phenotype category and are presented as medians with interquartile ranges (IQRs), as well as minimum and maximum values. Due to non-normal distribution of the quantitative data and unequal group sizes, non-parametric statistical methods were applied.

The association between Colorvue phenotype categories (ordinal variable) and ultrasonic gingival thickness values (continuous variable) was assessed using Kendall’s rank correlation coefficient (τ-b), which was selected due to the presence of tied ranks within the qualitative classification.

Differences in ultrasonic gingival thickness among the four Colorvue phenotype categories (thin, medium, thick, and very thick) were analyzed using the Kruskal–Wallis test. When a statistically significant overall difference was identified, post hoc pairwise comparisons were performed using Dunn’s test with Bonferroni correction.

In addition, descriptive thickness ranges associated with individual Colorvue phenotype categories were derived based on the distribution of ultrasonic measurements, including medians and interquartile ranges. These ranges were intended as empirical, method- and site-specific reference values rather than predefined diagnostic thresholds. Additionally, receiver operating characteristic (ROC) curve analysis was performed to evaluate the ability of ultrasonic gingival thickness measurements to discriminate between thin and non-thin phenotypes, as defined by dichotomized Colorvue probe classification. The area under the ROC curve (AUC) was calculated as a measure of discriminatory performance.

All statistical analyses were performed using R statistical software (R version 4.5.2, R Foundation for Statistical Computing, Vienna, Austria). A two-tailed significance level of α = 0.05 was applied.

## 3. Results

### 3.1. Study Sample and Measurements

A total of 92 consecutive patients presenting for screening were initially assessed for eligibility. At this stage, patients with active periodontal disease or clinical signs of gingival inflammation accompanied by bleeding on probing were excluded (*n* = 12). The remaining 80 patients underwent further screening, including assessment of systemic health status and radiological evaluation. All 80 patients who passed the initial periodontal screening were included in the present methodological diagnostic substudy and underwent standardized non-invasive soft tissue thickness assessment during the qualification visit. Of these, 36 patients fulfilled all predefined inclusion criteria and were subsequently enrolled in the parent randomized clinical trial.

For the purposes of the present methodological validation analysis, 80 patients aged between 18 and 60 years were included. Each patient contributed one standardized measurement site located at the tooth adjacent to the edentulous space, resulting in a total of 80 measurement sites.

### 3.2. Association Between Colorvue Phenotype Classification and Ultrasonic Measurements

A strong and statistically significant monotonic association was observed between qualitative gingival phenotype classification obtained using the Colorvue biotype probe and quantitative gingival thickness values measured with ultrasonography.

Given the ordinal nature of the Colorvue gingival phenotype categories and the presence of tied ranks, Kendall’s rank correlation coefficient (τ-b) was applied. The analysis demonstrated a strong positive correlation between Colorvue phenotype category and ultrasonic gingival thickness (τ = 0.71), which was highly statistically significant (z = 8.32, *p* < 0.001). These results indicate that higher Colorvue phenotype categories consistently correspond to increased gingival thickness values measured with the Pirop ultrasound-based system.

### 3.3. Comparison of Ultrasonic Gingival Thickness Across Colorvue Categories

Quantitative gingival thickness values measured with ultrasonography differed significantly among the four Colorvue phenotype categories. The Kruskal–Wallis test revealed a highly significant overall difference in gingival thickness between groups (χ^2^ = 55.66, df = 3, *p* < 0.001).

Post hoc pairwise comparisons performed using Dunn’s test with Bonferroni correction demonstrated that statistically significant differences were primarily observed between non-adjacent phenotype categories, particularly between thin and thick or very thick phenotypes. In contrast, partial overlap was observed between adjacent categories, such as medium and thick phenotypes. This pattern reflects gradual biological transitions in gingival thickness rather than sharp categorical boundaries and is consistent with the qualitative, screening-oriented nature of probe-based phenotype assessment.

**Figure 4:**
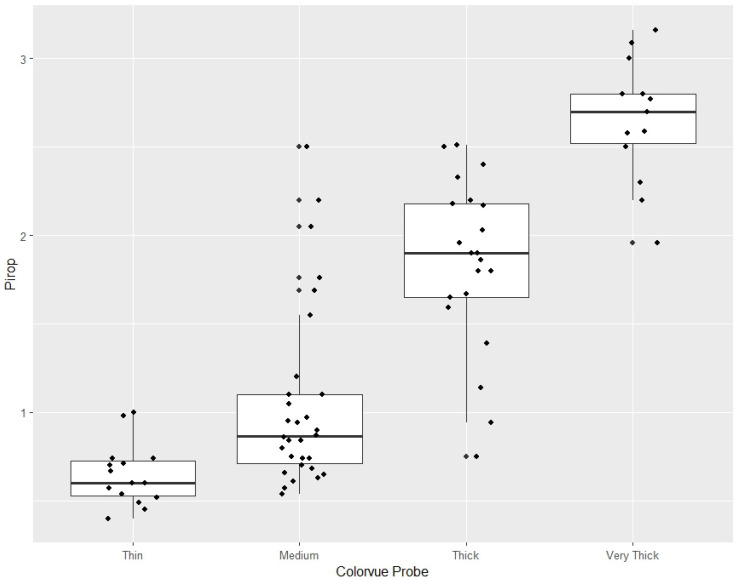
Distribution of ultrasonic gingival thickness values obtained with the Pirop system across Colorvue phenotype categories. The figure demonstrates a monotonic increase in gingival thickness with increasing phenotype category, while illustrating expected overlap between adjacent qualitative groups.

The distribution of ultrasonic gingival thickness values across Colorvue phenotype categories is illustrated in Figure 4. The box-and-whisker plot demonstrates a clear stepwise increase in median gingival thickness from thin to very thick phenotypes, with increasing variability in thicker categories and partial overlap between adjacent groups.

When Colorvue assessment was dichotomized into thin versus non-thin phenotypes, ultrasonic measurements demonstrated high discriminatory performance (AUC ≈ 0.91), supporting the use of probe-based translucency assessment as an effective screening method for identifying thin gingival tissues.

Overlap of ultrasonic thickness values was mainly observed between adjacent categories (medium and thick), whereas thin and very thick phenotypes showed minimal distributional overlap

### 3.4. Ultrasonic Gingival Thickness Distribution According to Colorvue Phenotype Classification

Descriptive analysis of ultrasonic measurements revealed that individual Colorvue gingival phenotype categories were associated with distinct, although partially overlapping, ranges of gingival thickness values. These ranges reflect empirical distributions observed under routine clinical conditions rather than predefined diagnostic thresholds.

Thin phenotypes assessed using the Colorvue probe were predominantly associated with low ultrasonic gingival thickness values, clustering below approximately 0.6 mm. Medium phenotypes demonstrated intermediate thickness values, most commonly ranging between approximately 0.6 and 1.0 mm. Thick phenotypes were associated with substantially higher ultrasonic gingival thickness values, typically exceeding approximately 1.5 mm. Very thick phenotypes corresponded to the highest ultrasonic measurements, most frequently above approximately 2.4 mm.

Although partial overlap was observed between adjacent phenotype categories—particularly between medium and thick phenotypes—a clear stepwise increase in gingival thickness across Colorvue categories was evident (Table 1). These findings are consistent with the qualitative and screening-oriented nature of probe-based phenotype assessment and support the interpretation of Colorvue classification as a tool for stratifying gingival tissues into clinically meaningful thickness ranges rather than providing exact quantitative measurements.

## 4. Discussion

The present methodological validation study evaluated the correspondence between qualitative gingival phenotype assessment performed using a color-coded periodontal probe and quantitative gingival thickness measurements obtained with a non-invasive ultrasound-based system. This observational substudy was embedded within a randomized clinical trial framework, enabling standardized data collection while remaining independent of treatment allocation, and allowing validation of the ultrasound-based measurement approach under routine clinical conditions. A strong and statistically significant monotonic association between Colorvue phenotype categories and ultrasonic gingival thickness values was observed, supporting the role of color-coded probes as clinically useful screening tools rather than precise quantitative instruments.

The 2017 World Workshop on the Classification of Periodontal and Peri-Implant Diseases and Conditions [17] provides a simplified thin–thick classification of gingival phenotype for clinical decision-making; however, it does not specify a standardized anatomical landmark for gingival thickness measurement. As a result, considerable heterogeneity exists in the literature regarding the reference level used for quantitative assessment. To ensure methodological consistency and direct comparability between qualitative and quantitative assessments, gingival phenotype evaluation using a color-coded periodontal probe and ultrasound-based gingival thickness measurements were performed at the same anatomical level. In the present study the color-coded probe was inserted into the gingival sulcus to a depth of approximately 2 mm during translucency assessment; ultrasound measurements were obtained at the corresponding sulcular level to reflect the clinically relevant site of probe-based phenotype assessment and to minimize landmark-related variability between measurement techniques.

From a clinical perspective, gingival phenotype assessment is primarily applied for risk stratification and treatment planning in procedures in which soft tissue thickness may influence outcomes, including implant placement, periodontal and mucogingival surgery, and orthodontic treatment. In this context, the binary thin–thick classification recommended by the 2017 World Workshop represents a simplified clinical framework rather than a strict biological dichotomy, allowing for more granular qualitative stratification in routine practice [17].

Probe translucency–based phenotype assessment has previously been investigated in preclinical models. Fischer et al. proposed gingival thickness ranges corresponding to qualitative phenotype categories using an agreement-based threshold approach in an ex vivo setting, defining thin gingiva as <0.5 mm, moderate as 0.5–0.8 mm, and thick as >0.8 mm. Although absolute thickness values identified in the present clinical study were higher, a consistent monotonic relationship between increasing phenotype category and increasing tissue thickness was observed, in line with the conceptual framework proposed by Fischer et al.

The observed differences in absolute thickness values are likely attributable to methodological factors, including in vivo versus ex vivo conditions, tissue perfusion, anatomical measurement site, and the use of ultrasonography as a quantitative reference method. Importantly, both studies demonstrated partial overlap between adjacent phenotype categories, supporting the interpretation of gingival phenotype as a biological continuum rather than a set of sharply defined classes.

Taken together, the present findings complement previous preclinical observations by confirming that color-coded probe-based phenotype assessment reliably stratifies gingival tissues into clinically meaningful thickness ranges, while highlighting the method- and context-dependent nature of absolute gingival thickness thresholds.

Different reference values for gingival phenotype categories have been reported by Aslan et al. in a preliminary clinical study evaluating the correlation between color-coded phenotype probes and soft tissue thickness measured using cone-beam computed tomography (CBCT) [20]. Using CBCT as the reference method, the authors proposed higher cutoff values for phenotype differentiation. Similarly, Sabri et al. demonstrated that the traditionally accepted 1.0 mm threshold for distinguishing thin and thick gingiva may not provide optimal diagnostic accuracy and proposed a higher cutoff value of 1.18 mm when histology was used as the reference standard [21]. Guliyev et al. reported clinically relevant gingival thickness thresholds of ≤0.92 mm and ≤0.80 mm for distinguishing thin gingival biotypes. Although color-coded probes showed acceptable performance in identifying thin phenotypes, their diagnostic accuracy markedly decreased when applied to the differentiation of medium and thicker gingival categories [22].

With respect to quantitative assessment, studies evaluating ultrasonic gingival thickness measurement using Measurement System Analysis have demonstrated acceptable repeatability and reproducibility, confirming the reliability of ultrasonography as a measurement technique [23]. Dziewulska et al. used ultrasonography solely as a reference method without defining diagnostic cutoff values and concluded that both visual assessment and the probe transparency method are reliable qualitative tools for gingival phenotype assessment in the maxillary anterior region [24]. A recent systematic review by Barausse et al. summarized the available clinical evidence on the diagnostic applications of ultrasonography in implant dentistry, demonstrating its reliability for the assessment of peri-implant soft tissue thickness, tissue phenotype, ridge morphology, and postoperative monitoring when compared with CBCT and clinical measurements [25]. However, the authors emphasized substantial heterogeneity in study designs, ultrasound devices, and imaging protocols [25]. Soltani et al. reported no significant differences between ultrasonographic and direct clinical measurements of gingival thickness in the anterior region, whereas a minor but statistically significant underestimation was observed at posterior sites [26]. This discrepancy was primarily attributed to technical factors related to probe positioning and tissue characteristics in less accessible regions. Importantly, earlier work by Furtak et al. demonstrated that accurate ultrasonic measurements require strict control of probe pressure, which should not exceed 25 g, as even minimal tissue compression may influence recorded thickness values [23]. This observation is consistent with our clinical experience, highlighting the need for extremely gentle probe contact during ultrasound-based soft tissue assessment.

Differences in cutoff values across studies further emphasize the method-dependent nature of gingival phenotype thresholds. Variations in reference methods, measurement locations, and study design inherently influence absolute thickness values and derived cutoffs. Despite these differences, Sabri et al. [21] demonstrated that color-coded probe-based gingival phenotype assessment provides high diagnostic accuracy for qualitative classification of gingiva as thin or thick. Importantly, the authors emphasized that the diagnostic performance of probe transparency methods improves when phenotype categories are simplified to a binary classification, supporting their primary role as screening tools rather than precise quantitative instruments.

Furthermore, Sabri et al. reported that ultrasonography demonstrated the highest agreement and the lowest systematic bias among all non-invasive techniques evaluated, outperforming both CBCT and transgingival probing. These findings support the use of ultrasonography as the most reliable non-invasive quantitative method for gingival thickness assessment, particularly in clinical scenarios requiring repeated measurements or longitudinal monitoring.

Importantly, despite methodological heterogeneity across studies, a consistent association between color-coded probe-based phenotype classification and quantitative measures of soft tissue thickness has been demonstrated. This convergence supports the role of color-coded probes as clinically useful screening tools, while underscoring the need for method-specific validation and complementary quantitative assessment when interpreting absolute thickness ranges and diagnostic accuracy.

Recent evidence from Bolat and Lutfioglu further highlights that gingival phenotype classification is highly dependent on the anatomical level at which gingival thickness is assessed, demonstrating that measurements obtained at different vertical locations may lead to different phenotype assignments [27]. Importantly, these findings indicate that gingival thickness measured at a single site does not necessarily reflect tissue thickness at adjacent locations, such as more apical vestibular areas, edentulous sites, or even other teeth within the same patient. Consequently, gingival phenotype should be regarded as a site-specific rather than a patient-wide characteristic. Within this context, the measurement strategy applied in the present study—based on a clearly defined and clinically relevant anatomical level—addresses an important source of variability reported in the literature.

From a clinical standpoint, the findings of the present study strongly support a complementary diagnostic approach in which color-coded periodontal probes serve as rapid and feasible screening tools during initial patient assessment, while ultrasound-based measurements provide objective, reproducible, and non-invasive quantitative data required for informed treatment planning and reliable longitudinal monitoring.

While several studies have proposed different gingival thickness thresholds using heterogeneous reference methods, the present study offers ultrasound-derived quantitative ranges obtained under routine clinical conditions and directly aligned with the probe-based assessment site. Rather than aiming to define universal or definitive cutoff values, the thresholds proposed here should be interpreted as clinically meaningful and practically applicable references to support everyday clinical decision-making.

The tables (Table 2 and Table 3) present a comparative evaluation of Colorvue probe–based and Pirop ultrasound–based soft tissue measurements, alongside a synthesis of gingival thickness thresholds proposed in the literature for phenotype classification.

Several limitations of the present methodological validation study should be acknowledged. First, inter- and intra-rater reliability were not assessed, as all measurements were performed by a single calibrated examiner and the primary objective was comparison between qualitative probe-based assessment and quantitative ultrasound measurements rather than an evaluation of device reproducibility. Second, gingival phenotype was assessed at localized measurement sites.

## 5. Conclusions

Within the limitations of this methodological validation study, qualitative gingival phenotype assessment using a color-coded periodontal probe demonstrated clinically meaningful correspondence with ultrasound-derived soft tissue thickness ranges.

Color-coded probes appear suitable for rapid chairside screening and initial risk stratification, whereas ultrasonography provides a reliable, non-invasive method for quantitative assessment and longitudinal monitoring of gingival thickness at defined anatomical sites.

The findings support a complementary diagnostic framework in which qualitative phenotype classification and quantitative ultrasound measurement serve distinct but synergistic roles in contemporary clinical practice.

The ultrasound-derived thickness ranges presented in this study may serve as clinically useful reference values for the interpretation of probe-based phenotype assessment, while acknowledging that absolute cutoff values remain method- and site-dependent.

## Figures and Tables

**Table 1:** Ultrasonic gingival thickness ranges associated with Colorvue phenotype categories.

Colorvue Phenotype	Predominant Ultrasonic Thickness Range (mm) *
Thin	<~0.6
Medium	~0.6–~1.0
Thick	>~1.5
Very thick	>~2.4

* Values represent approximate ranges derived from medians and interquartile distributions and should be interpreted as descriptive reference ranges rather than diagnostic thresholds.

**Table 2:** Reported gingival thickness thresholds for phenotype classification, in the literature.

Study	Study Design	Reference Method	Phenotype Categories	Reported Threshold Values (mm)	Key Remarks
Fischer et al. [15]	Preclinical, ex vivo	Direct thickness measurement	Thin/Moderate/Thick/Very thick	Thin < 0.5, Moderate 0.5–0.8, Thick/Very thick > 0.8	Controlled conditions; absence of perfusion; lower thresholds
Aslan et al. [20]	Clinical, in vivo	CBCT	Thin/Medium/Thick/Very thick	Thin–Medium ≈ 0.83, Medium–Thick ≈ 1.07, Thick–Very thick ≈ 1.24	ROC-based cutoffs; CBCT associated with higher values
Sabri et al. [21]	Diagnostic accuracy study (cadaver heads)	Histology (gold standard)	Thin/Thick (binary)	Thin–Thick = 1.18	Binary classification improves accuracy; research-oriented cutoff
Guliyev et al. [22]	Clinical, in vivo	Transgingival measurement (EF, FP)	Thin vs. non-thin	≤0.92; ≤0.80	Color-coded probes effective for thin phenotype detection; limited discrimination of thicker categories
Present study	Clinical, in vivo	Ultrasonography (Pirop)	Thin/Moderate/Thick/Very thick	Thin < ~0.6, Moderate ~0.6–~1.0, Thick > ~1.5, Very thick > ~2.4	Thresholds derived using ultrasound; method-dependent values

**Table 3:** Comparison between Colorvue biotype probe and Pirop ultrasound-based measurement for oral soft tissue assessment.

Parameter	Colorvue Biotype Probe	Pirop Ultrasound-Based Measurement
**Principle of measurement**	Visual assessment based on probe translucency through gingival tissues; phenotype classification	Ultrasonic A-scan measurement based on sound wave reflection; direct tissue thickness quantification
**Type of outcome**	Qualitative/categorical (thin, medium, thick phenotype)	Quantitative (continuous values in mm)
**Measurement accuracy**	Limited; does not provide exact tissue thickness values	High accuracy and resolution; provides precise numerical measurements
**Objectivity**	Partially subjective; dependent on light, tissue translucency and examiner interpretation	Objective digital measurement with reduced operator-dependent variability
**Invasiveness**	Minimally invasive probing	Completely non-invasive
**Patient comfort**	Generally well tolerated but involves probing	High patient comfort; no tissue penetration or anesthesia required
**Repeatability and monitoring over time**	Limited applicability for longitudinal monitoring	Highly suitable for repeated measurements and longitudinal follow-up
**Clinical applications**	Chairside gingival phenotype screening	Chairside, detailed clinical assessment, treatment planning, and monitoring
**Research applicability**	Limited; primarily descriptive or screening tool	High; enables standardized, reproducible measurements across studies
**Versatility**	Mainly phenotype classification	Broad applicability in implant dentistry, periodontology, orthodontics, and biomaterials research
**Key limitations**	Categorical output; lack of quantitative precision	Requires dedicated equipment and operator training

## Data Availability

The original contributions presented in the study are included in the article, further inquiries can be directed to the corresponding author.
